# Impacts of Pretransplant Infections on Clinical Outcomes of Patients with Acute-On-Chronic Liver Failure Who Received Living-Donor Liver Transplantation

**DOI:** 10.1371/journal.pone.0072893

**Published:** 2013-09-02

**Authors:** Kuo-Hua Lin, Jien-Wei Liu, Chao-Long Chen, Shih-Hor Wang, Chih-Che Lin, Yueh-Wei Liu, Chee-Chien Yong, Ting-Lung Lin, Wei-Feng Li, Tsung-Hui Hu, Chih-Chi Wang

**Affiliations:** 1 Liver Transplantation Program and Department of Surgery, Kaohsiung Chang Gung Memorial Hospital, Department of Internal Medicine, Kaohsiung Chang Gung Memorial Hospital, Kaohsiung, Taiwan; 2 Department of General Surgery, Changhua Christian Hospital, Changhua, Taiwan; 3 Division of Infectious-Diseases, Department of Internal Medicine, Kaohsiung Chang Gung Memorial Hospital, Kaohsiung, Taiwan; 4 Chang Gung University College of Medicine, Kaohsiung, Taiwan; 5 Division of Hepato-Gastroenterology, Department of Internal Medicine, Kaohsiung Chang Gung Memorial Hospital, Kaohsiung, Taiwan; The University of Hong Kong, Hong Kong

## Abstract

**Background:**

Liver transplantation is the only therapeutic modality for patients with acute-on chronic liver failure (ACLF). These patients are at high risk for bacterial infections while awaiting transplantation. The aim of this study was to elucidate whether an adequately treated bacterial infection influences the outcomes after transplantation in this patient population.

**Methodology/Principal Findings:**

54 recipients (median age, 49.5 years [range, 22–60]) of adult-to-adult living donor liver transplant (LDLT) for ACLF were categorized as those with pretransplant infection (Group 1, n = 34) or without pretransplant infection (Group 2, n = 20) for retrospective analyses. With the exception of a higher male-female ratio (*P* = 0.046) and longer length of pretransplant hospital stay (*P* = 0.026) in Group 1, similar demographic, laboratory and clinical features were found in both groups. Patients in Group 1 (totally 42 pretransplant infection episodes) were adequately treated with effective antibiotic(s) before receiving LDLT. All included patients were followed up until one year after transplantation or death. Sixty-one posttransplant infection episodes were found in an overall of 44 ACLF patients (27 in Group 1 vs. 15 in Group 2; *P* = 0.352). Frequently encountered posttransplant infections were intraabdominal infection, pneumonia, bloodstream infection and urinary tract infection. Two patients died in each group (*P* = 0.622). No significant difference was found in the length of posttransplant ICU stay, and in one-year survival, graft rejection, and posttransplant infection rate between both groups. The longer overall hospital stay (mean day, 89.0 vs. 65.5, *P* = 0.024) found in Group 1 resulted from a longer pretransplant hospital stay receiving treatment for pretransplant infection(s) and/or awaiting transplantation.

**Conclusions:**

These data suggested that an adequately treated pretransplant infection do not pose a significant risk for clinical outcomes including posttransplant fatality in recipients in adult-to-adult LDLT for ACLF.

## Introduction

Liver transplantation is the only curative modality for patients with relentless liver failure resulting from chronic hepatic decompensation or acute exacerbation of chronic liver disease. Among liver transplant recipients, a substantial number of patients have been of cases of acute-on-chronic liver failure (ACLF) [1]. The prognosis is dismal when such patients develop multiorgan failure. Of note, ACLF patients receiving supportive treatment while awaiting liver transplant are at high risk for bacterial infections [1], [2], and data regarding whether an adequately treated bacterial infection influences the outcomes after transplantation in this patient population are scarce. The aim of this study was to elucidate the impact of pretransplant infection in patients with ACLF on the outcomes after liver transplantation in general, and posttransplant infections and mortality in particular.

## Patients and Methods

### Hospital Setting

We retrospectively reviewed ACLF patients who underwent adult-to-adult living donor liver transplant (LDLT) at Kaohsiung Chang Gung Memorial Hospital between January 2001 and December 2009. This study was conducted with a waiver of patient consent approved the Institutional Review Board of Chang Gung Memorial Hospital (Document no. 101–0564B).

### Definitions

ACLF was defined as an acute hepatic insult manifesting as jaundice (serum bilirubin >5 mg/dl) and coagulopathy (international normalized ratio >1.5) complicated within 4 weeks by ascites and/or encephalopathy in a patient with previously diagnosed or undiagnosed chronic liver disease [3]. All patients were followed up until one year after liver transplant or death. Ascites was detected by sonography and/or findings at laparotomy. Hepatic encephalopathy was clinically diagnosed based on neurologic findings, and its severity was stratified according to the West Haven Criteria [4]. The included ACLF patients were categorized as those with a pretransplant infection or those without a pretransplant infection. Preoperative management of these patients included supportive care for liver failure, and antiviral treatment for viral hepatitis, antibiotic therapy for bacterial infection(s), and/or mechanical ventilatory support for respiratory failure as necessary.

A pretransplant infection referred to an infection occurred within 4 weeks prior to liver transplantation, which was diagnosed based on clinical and laboratory (e.g., blood, ascites, sputum, and urine) and/or imaging (e.g., chest X-ray, ultrasonography, and CT) findings. Pretransplant infections were categorized into bloodstream infection (BSI), pneumonia, spontaneous bacterial peritonitis (SBP), and urinary tract infection (UTI). Sepsis was defined as an infection with the presence of systemic inflammatory response syndrome in the affected patient, which was manifested by two or more of the following conditions: (i) temperature >38°C or <36°C, (ii) heart rate >90 beats/min, (iii) respiratory rate >20 breaths/min or PaCO_2_ <32 mmHg, and (iv) peripheral white cell count (WBC)> 12,000/ mm^3^, <4000/ mm^3^, or consisting of>10% immature (band-form) cell [5]. BSI was defined as an infection with pathogen(s) isolated from blood culture. A microbe of normal commensals (e.g., coagulase-negative staphylococci and Gram-positive bacilli) was regarded as a significant pathogen for BSI if (i) it grew from ≥2 blood specimens sampled from different sites at different time points, or (ii) it grew from only one blood specimen of a clinically septic patient with a predisposing factor (e.g., central catheterization and/or indwelling foreign device) for infection due to this pathogen [6], and his or her sepsis improved after receiving antibiotic therapy accordingly, with or without removal of a related catheter and/or device. BSI referred to a significant bacteremia in a patient from whom no apparent infection focus was found; otherwise, the bacteremia was regarded as one secondary to the infection focus. Pneumonia was defined as the emergence of a new infiltrate or presence of a progressive infiltrate on chest radiography of a septic patient with fever, leukocytosis, purulent sputum or increased respiratory secretions, emerging or worsening cough, dyspnea, tachycardia, rales detected at auscultation and/or arterial oxygen desaturation [6], [7]. UTI was defined as the presence of dysuria, frequency and/or urgency with or without flank knocking pain in a septic patient with pyuria. SBP was defined as the presence of a peritoneal sign such as muscle guarding and/or rebound tenderness over the distended abdomen of a patient with a polymorphonuclear neutrophil count ≥250 cells/mm^3^ in his or her ascites, regardless bacterial growth from culture [8]. Bacterial isolates were identified by conventional methods and/or the automated ID 32GN system (Vitek Systems, bioMérieux, Hazelwood, MO). Antibiotic susceptibility testing was performed on a daily-service basis using Kirby-Bauer disk-diffusion methods [9] and interpretive criteria for susceptibility recommended by CLSI (formerly NCCLS) [10].

### Medical Management

All patients with a diagnosed bacterial infection were given antibiotic(s) on empirical basis, which was then adjusted based on recommendations made by an infectious-disease specialist if the infection clinically deteriorated, or was subsequently switched to other antimicrobial(s) as was indicated by the results of culture and susceptibility testing. A pretransplant bacterial infection was considered adequately treated and the affected patient was regarded as an eligible transplant recipient only when he or she fulfilled the criteria as follows: (i) disappearance of symptoms and signs suggestive of sepsis, and (ii) normalization or improvement of laboratory and/or imaging findings indicating bacterial infection.

### Liver Transplantation and Postoperative Care

Hepatectomy in donors and recipients and liver engrafting for recipients in LDLT were performed as described previously [11]–[13]. The majority of liver grafts were harvested from the right lobe, with or without outflow reconstruction for connecting the branches of the middle hepatic vein. All patients received immunosuppressants that include tacrolimus, low-dose steroid, and mycophenolate mofetil as standard immunosuppressant regimen. The dosing of these immunosuppressants was adjusted based on the function of the liver graft. As for prophylaxis for postoperation bacterial and *Candida* infections, combination of either cefepime or ceftazidime (20 mg/kg/8 hours) and teicoplanin (400 mg/day) was started immediately before operation and lasted for 3–5 days depending upon posttransplant conditions, and fluconazole 100mg/ day was started after transplantation and was used during post-operation stay at ICU. Daily trimethoprim 80mg/sulfamethoxazole 400 mg was started after operation and was used indefinitely as prophylaxis for *Pneumocystis jiroveci*. Ganciclovir (5 mg/kg/12 hours) was used when cytomegalovirus (CMV) infection was clinically suspected or upon detection of CMV antigenemia.

All recipients were admitted to Liver Intensive Care Unit (LICU) for postoperative care, where laboratory data (mainly hemogram and blood chemistry) were closely monitored. Doppler ultrasound was performed daily to check the vascular flow in the graft for one week after liver transplant and then twice weekly until the patient was discharged. These patients were additionally evaluated by a dedicated infectious-disease specialist (Dr. JW Liu) on daily basis. Once bacterial infection was suspected, empirical antibiotic therapy immediately started.

### Statistical Analyses

Data of the included ACLF patients were retrieved from their medical records. Between different patient groups, comparison of categorical variables was carried out using the *χ* ^2^ test or Fisher exact test when appropriate, and comparison of continuous variables was performed using *t*-test or Mann-Whitney *U* test, where applicable. To ascertain the impact of pretransplant infection on the postoperative mortality by eliminating potential confounding(s), pretransplant infection and other variables between fatal and survived liver transplant recipients were entered into a multivariate analysis using a logistic regression model. A *P* value <0.05 was considered statistically significant. All statistical analyses were performed using SPSS for Windows, version 16.0 (SPSS Inc, Chicago, IL, USA).

## Results

### Demographic, Clinical and Laboratory Features of ACLF Patients

Among a total of 408 patients received adult-to-adult LDLT during the study period, 54 (36 men and 18 women [median age = 49.5 years, range = 22–60 years]) were found to be of ACLF cases and were included for analyses. None of these patients was put on an artificial liver support system (ALSS) prior to liver transplant. The included ACLF patients were categorized as those with pretransplant infection (Group 1, n = 34) or those without pretransplant infection (Group 2, n = 20). With the exception of a higher male-female ratio (26∶8 vs. 10∶10, *P* = 0.046) and longer length of pretransplant hospital stay (mean, 28.0 vs. 14.5 days; *P* = 0.026) in Group 1 than in Group 2, similar demographic, laboratory and clinical features were found in both groups, and hepatitis B was the most commonly encountered liver disease among the overall included ACLF patients (Table 1). The median of MELD (model for end-stage liver disease) score was 24 for both Group 1 and Group 2 [14]. No significant difference was found in the lengths of pretransplant hospital stay (31.9±23.8 vs. 21.5±20.6 days; *P* = 0.149) and overall hospital stay (85.9±30.4 vs. 74.82±27.2 days; *P* = 0.948) between patients with MELD scores≧25 and those with MELD scores <24 among the overall included patients.

**Table 1:** Demographic, clinical and laboratory features of 54 patients with acute-on-chronic liver failure with or without pre-transplant infection.

Demographic, clinical or laboratory feature	Group 1 (with pre-transplant infection) N = 34	Group 2 (without pre-transplant infection) N = 20	*P* value
Gender (Male: Female)	26 : 8	10 : 10	**0.046**
Age [year; median (range)]	49 (30–60)	50.5 (22–58)	0.914
Child-Pugh score, median (range)	12 (9–15)	12 (8–15)	0.701
MELD score, median (range)	24 (16–39)	24 (19–40)	0.440
Serum total bilirubin [mmol/L; median (range)]	12.05 (5.1–45.2)	17.05 (5–64.4)	0.993
Serum albumin [g/dL L; median (range)]	2.6 (1.8–3.4)	2.5 (1.7–3.6)	0.535
Prothrombin time			
Second, median (range)	17.25 (14.9–50)	18.55 (15.1–50)	0.286
INR, median (range)	1.71 (1.5–5)	1.84 (1.51–5)	0.230
Pretransplant status			
ICU stay, n (%)	10 (29.4)	7 (35)	0.672
ICU stay [day; median (range)]	0 (0–25)	0 (0–30)	0.456
Hospital stay [day; median (range)]	28 (2–85)	14.5 (2–57)	**0.026**
Underlying liver disease, n (%)			0.423
Hepatitis B	23 (67.6)	12 (60.0)	
Hepatitis C	3 (8.8)	1 (5.0)	
Alcoholic liver disease	5 (14.7)	2 (10.0)	
Metabolic liver disease	3 (8.8)	5 (25.0)	
Pretransplant hepatic encephalopathy n (%)			0.335
Grade I & II			
Grade III & IV	4 (40.0)	5 (71.4)	
	6 (60.0)	2 (28.6)	
Liver graft			
GRWR ≧0.8, n (%)	29 (85.3)	17 (85.0)	0.977
Right lobe	32 (94.1)	19 (95.0)	1.000
Outflow reconstruction	22 (64.7)	11 (55.0)	0.480
Age of donor, median year (range)	28 (18–47)	28.5 (19–48)	0.554
Intraoperative blood loss, mean (mL±SD)	12006.1±15515.6	6040.8±9539.2	0.054
Transfused blood, mean (mL±SD)	8738.7±11011.8	5000.39±6388.0	0.119

Abbreviations: SD = Standard deviation; MELD = Model for end-stage liver disease; INR = International normalized ratio; ICU = Intensive-care unit; GRWR: graft-to-recipient weight ratio.

Figure in bold font indicates a significant statistical difference.

### Pretransplant Infections

A total of 42 pretransplant infection episodes were found in the 34 LDLT recipients. Among these patients, 26 (76.5%) experienced one episode of pretransplant infection, while the rest 8 each experienced 2. Major pretransplant infection entities in decreasing order were UTI (n = 14), SBP (n = 11), pneumonia (n = 7) and BSI (n = 6); pretransplant infection entities and the pathogens are summarized in Table 2. The most frequently found pathogens were *Klebsiella pneumoniae* (n = 7). All patients with pretransplant infection received effective antibiotic therapy, and extracorporeal shock-wave lithotomy was additionally carried out for one patient with urolithiasis suffering urinary tract infection. The mean time lapse from starting an antimicrobial treatment for pretransplant infection to LDLT was 13.8±12.6 days.

**Table 2:** Episodes of pretransplant infection (N = 42) found in 34 living-donor liver transplant recipients.

Infection entity and pathogen(s)	Episode
Spontaneous bacterial peritonitis*, % (N/**N**)	26.2 (11/42)
Without bacteremia, % (n_1_/**N**)	19 (8/42)
Culture-negative neutrocytic ascites, % (n_2_/n_1_)	87.5 (7/8)
*Pseudomonas* species, % (n_2_/n_1_)	12.5 (1/8)
With secondary bacteremia, % (n_1_/**N**)	7.1 (3/42)
* Klebsiella pneumoniae*, % (n_2_/n_1_)	100 (3/3)
Primary bloodstream infection, % (N/**N**)	14.3 (6/42)
* Klebsiella pneumoniae*, % (n/N)*	66.7 (4/6)
* Staphylococcus aureus*, % (n/N)	16.7 (1/6)
* Corynebacterium* species, % (n/N)	16.7 (1/6)
Pneumonia*, % (N/**N**)	15.9 (7/44)
Urinary tract infection, % (N/N)	33.3 (14/42)
Without bacteremia, % (n_1_/**N**)	31 (13/42)
* Escherichia coli*, % (n_2_/n_1_)	7.1 (1/14)
* Pseudomonas* species‡, % (n_2_/n_1_)	7.1 (1/14)
Gram-positive bacilli†, % (n_2_/n_1_) †	28.6 (4/14)
Gram-negative bacilli†, % (n_2_/n_1_)	28.6 (4/14)
* Candida* species‡, % (n_2_/n_1_)	7.1 (1/14)
Pathogens not identified, % (n_2_/n_1_)	28.6 (4/14)
With bacteremia, % (n_1_/**N**)	2.4 (1/42)
* Escherichia coli*, % (n_2_/n_1_)	100 (1/1)
Cellulitis, % (N/**N**)	4.8 (2/42)
Other infections§, % (N/**N**)	4.8 (2/42)
Episodes with the pathogen isolated, % (N/**N**)	47.6 (20/42)

* Concurrent spontaneous bacterial peritonitis and pneumonia were found in one recipient.

† Isolates of Gram-positive bacillus and Gram-negative bacillus were identified as co-pathogens in one episode of urinary tract infection.

‡ Isolates of *Pseudomonas* species and *Candida* species were identified as co-pathogens in one episode of urinary tract infection.

§ Including septic arthritis caused by *Pseudomonas stutzeri*, and acute cholecystitis each one episode.

### Outcomes after Liver Transplantation

With the exception of a longer total hospital stay (89.0 days [range, 30–163] vs. 65.5 days [44–117], *P* = 0.024) in Group 1 than in Group 2, no significant difference was found in the length of posttransplant ICU stay, and in one-year survival, graft rejection, and posttransplant infection rates between both groups (Table 3). Two fatal cases were found in each group, accounting for a fatality rate of 6.0% in Group 1 and of 10.0% in Group 2, respectively (*P* = 0.622). In Group 1, one patient experienced posttransplant hepatic arterial thrombosis and died of intractable internal bleeding with hypovolumic shock and intraabdominal *Candida* infection 5 days after retransplantation, while the other died of progressive multiorgan failure one day after LDLT. In Group 2, one patient who experienced biliary leak after LDLT died of biliary tract infection with sepsis 3 months later, while the other who experienced posttransplant graft failure with persistent coagulopathy and internal bleeding died of hepatic failure, pneumonia and bacterial peritonitis with sepsis one month after liver transplantation. Multivariate analysis disclosed that none of the evaluated variables including the length of posttransplant hospital stay, graft rejection, posttransplant infection, and pretransplant infection significantly differed (*P*>0.050) between fatal and survived liver transplant recipients.

**Table 3:** Outcomes after living-donor liver transplantation in recipients suffered acute-on-chronic liver failure with or without pretransplant infection.

	Group 1 (with pretransplantinfection) N = 34	Group 2 (withoutpretransplant infection) N = 20	*P* value
Post-liver transplant			
Length of ICU stay, median day (range)	21.5 (1–59)	22.5 (14–62)	0.851
Length of hospital stay, median day (range)	52 (1–123)	47 (28–94)	0.667
Overall ICU stay, median day (range)	24.5 (14–59)	23.5 (14–62)	0.667
Overall hospital Stay, median day (range)	89 (30–163)	65.5 (44–117)	**0.024**
Rejection (≦1 year after transplant), n (%)	6 (17.6)	4 (20)	1
1-year graft survival, n (%)	32 (94.1)	18 (90)	0.622
Patients with posttransplant infection, n (%)	28 (82.3)	16 (80)	0.831
Episode of posttransplant infection			
Intra-abdominal infection*	20	13	0.477
Pneumonia	10	5	0.727
Bloodstream infection	4	1	0.318
Urinary tract infection	4	2	1
1-year patient survival, n (%)	32 (94.1)	18 (90)	0.622

Abbreviation: ICU = Intensive-care unit.

Figure in bold font indicates a significant statistical difference.

* Including bacterial peritonitis, biliary tract infection, liver abscess and enterocolitis.

### Detailed Posttransplant Infections

A total of 61 posttransplant infection episodes were found in an overall of 44 ACLF patients (28 [82.4%] in Group 1 vs. 16 [80%] in Group 2; *P* = 0.352); these infection episodes and pathogens were detailed in Table 4. Bacterial peritonitis was the most frequently encountered posttransplant infection of which *Acinetobacter* species and coagulase-negative staphylococci were the leading pathogens. Intraabdominal infection was the most commonly found posttransplant infection regardless of pretransplant infection. Posttransplant infections developed in patients with pretransplant infection were summarized in Table 5, which in decreasing order were intraabdominal infection, pneumonia, primary BSI and UTI. Of note, one patient suffered pre- and post-liver transplant peritonitis caused by *Pseudomonas aeruginosa*, while another experienced pretransplant primary *K. pneumoniae* bacteremia and posttransplant peritonitis with a *K. pneumoniae* being isolated from ascites culture. Molecular differentiation was not performed to clarify whether these *P. aeruginosa* and *K. pneumoniae* isolates were of the same clone.

**Table 4:** Episodes of post-transplant infection (N = 61) found in 44 living-donor liver transplant recipients.

Infection category and pathogen(s)	Episode
Bacterial peritonitis, % (N/**N**)	36.1 (22/61)
Without bacteremia, % (n_1_/**N**)	34.4 (21/61)
Culture-negative neutrocytic ascites, % (n_2_/n_1_)	14.3 (3/21)
* Acinetobacter* species, % (n_2_/n_1_)*	28.6 (6/21)
Coagulase-negative *staphylococcus*, % (n_2_/n_1_)	23.8 (5/21)
* Enterobacter cloacae*, % (n_2_/n_1_)	9.5 (2/21)
* Enterococcus* species, % (n_2_/n_1_)	9.5 (2/21)
* Serratia marcescens*, % (n_2_/n_1_)	9.5 (2/21)
Other bacteria, % (n/N)†	19 (4/21)
* Candia* species, % (n_2_/n_1_)	4.8 (1/21)
With secondary bacteremia, % (n_1_/**N**)]	1.6 (1/61)
Coagulase-negative *Staphylococcus,* % (n_2_/n_1_)	100 (1/1)
Primary bloodstream infection, % (N/**N**)	8.2 (5/61)
Coagulase-negative staphylococci, % (n/N)	40 (2/5)
* Escherichia coli*, % (n/N)	20 (1/5)
* Staphylococcus hemolytic,* % (n/N)	20 (1/5)
* Aeromonas* species, % (n/N)	20 (1/5)
Pneumonia, % (N/**N**)	24.6 (15/61)
* Stenotrophomonas maltophilia*, % (n_1_/N)	20 (3/15)
Pathogens not identified, % (n_1_/N)	80 (12/15)
Urinary tract infection, % (N/**N**)	9.8 (6/61)
Without bacteremia, % (n_1_/**N**)	8.2 (5/61)
* Escherichia coli*, % (n_2_/n_1_)	20 (1/5)
* Klebsiella pneumoniae*, % (n_2_/n_1_)	20 (1/5)
* Candida* species, % (n_2_/n_1_)‡	20 (1/5)
Pathogens not identified, % (n_2_/n_1_)§	60 (3/5)
With bacteremia, % (n_1_/**N**)	1.6 (1/61)
* Escherichia coli*, % (n_2_/n_1_)	100 (1/1)
Biliary tract infection, (N/**N**)||	8.2 (5/61)
Liver abscess, (N/N)	3.3 (2/61)
Infectious colitis/enterocolitis, (N/**N**)¶	6.6 (4/61)
Cellulitis, % (N/**N**)	1.6 (1/61)
Surgical site infections, % (N/**N**)**	1.6 (1/61)
Episodes with pathogen(s) being isolated, % (N/**N**)	59 (36/61)

* Four episodes of bacterial peritonitis were simultaneously caused by 2 pathogens.

† Including *Pseudomonas* species, *Klebsiella pneumoniae*, *Stenotrophomonas maltophilia*, and *Prevotella bivia* each one isolate.

‡ One episode of urinary tract infection were simultaneously caused by 2 pathogens.

§ Including two episodes of urinary tract infection caused by Gram-positive coccus and one episode of clinically symptomatic pyuria; the pathogens were not identified on routine clinical-service based as the colony count was less than 10^5^/mL.

||Three episodes of biliary tract infection found to be caused by isolates of viridans streptococci (n = 2), *Enterococcus* species (n = 1), *Klebsiella pneumoniae* (n = 1) and one episode simultaneously caused by 2 pathogens.

¶ Including pseudomembranous colitis due to *Clostridium difficile* (n = 2), enterocolitis caused by *Salmonella enterica* (n = 1) and an enterocolitis episode of which the pathogen was unknown.

** Polymicrobial infection caused by *Pseudomonas* species, *Enterococcus faecalis* and *Corynebacterium* species.

**Table 5:** Posttransplant infection entities found in living-donor liver transplant recipients with pretransplant infections involving different organ systems*.

No. recipients and pretransplant infection episodes	Posttransplant infection entities (episode)			
	IAI†	BSI	pneumonia	UTI
Recipients with pretransplant UTI (n = 14)				
Episodes of posttransplant infection (n = 16)	7	2	5	2
Recipients with pretransplant SBP (n = 11)				
Episodes of posttransplant infection(n = 13)	7	1	4	1
Recipients with pretransplant pneumonia (n = 7)				
Episodes of posttransplant infection (n = 7)	4	0	3	0
Recipients with pretransplant primary BSI (n = 6)				
Episodes of posttransplant infection (n = 7)	4	1	1	1
Recipients with pretransplant cellulitis (n = 2)				
Episodes of posttransplant infection (n = 3)	2	0	0	1
Recipients with pretransplant septic arthritis (n = 1)				
Episodes of posttransplant infection (n = 2)	1	0	1	0

* One patient might experienced one or more pretransplant and/or posttransplant infections.

Abbreviations: UTI = Urinary tract infection, SBP = Spontaneous bacterial peritonitis, BSI = Bloodstream infection.

† Including bacterial peritonitis, biliary tract infection, liver abscess, and enterocolitis.

## Discussion

A total of the 49 pretransplant infection episodes developed in 34 (68%) of the overall 54 ACLF patients included in this series, and the most common pretransplant infection was UTI, as opposed to SBP that was reported to be the most frequently encountered pretransplant infection in patients with chronic liver failure [15]. ACLF features acute liver injury on top of chronic liver damage, and ACLF patients are subject to high chances of bacterial infection while awaiting liver transplantation. Among patients with chronic liver failure, the impacts of pretransplant infections on post-liver-transplant morbidity and mortality had been controversial before a recently published research by Sun et al. suggesting that an adequately treated pretransplant infection did not pose a significant risk for poor outcomes including posttransplant fatality [8].

In the circumstances of shortage of organ donated by a deceased donor, liver graft donated by a living adult shortens the waiting time for patients for whom urgent liver transplant is indicated [16]. Our report is the first series specifically addressed the impacts of pretransplant infection on posttransplant outcomes in ACLF patients who received LDLT, and the results suggest that ACLF patients with sufficiently treated pretransplant infection are not at higher risk of posttransplant mortality and morbidity. The longer length of overall hospital stay (mean, 89 vs. 65.5 days; *P* = 0.024) and similar lengths of posttransplant ICU and posttransplant hospital stay between patients with pretransplant infection and those without merely suggested that the former had a longer pretransplant hospital stay receiving treatment for their pretransplant infection and/or awaiting transplantation. Our data disclosed that in ACLF patients with an adequately treated pretransplant bacterial infection, irrespective of the anatomic site involved, frequently encountered posttransplant infections were intraabdominal infection, pneumonia, BTI and UTI, which were similar to those reported in other liver transplant series that included patients with chronic liver failure [2], [8], [17]–[19].

In case a liver graft not being available in a timely fashion, a plasma-exchange-centered ALSS was reported to be helpful in bridging ACLF to liver transplantation [20]. While it may downgrade the MELD score before liver transplantation, the ALSS is far from a salvage modality for ACLF [20]. Of note, ACLF patients being put on an ALSS are at high risk for bacterial infections as well [20], and the impact of a pretransplant infection on the post-liver-transplant outcomes in this patient population needs to be clarified by further studies.

ACLF *per se* compromises the immunity of the affected patients [21], and a major surgical operation and subsequently used immunosuppressants add to vulnerability to infections [19]. The risk for infection in liver transplant recipients depends on both microbial exposure and the immune-suppressed intensity, which are often referred to as the “net state of immunosuppression” [22]. Recipients are always subject to high chances of bacterial infections within months after liver transplantation [18], [19].

Of note, frequently seen pathogens for hospital-acquired infections such as *Acinetobacter baumannii*, *Serratia marcescens*, and *Stenotrophomonas maltophilia* [23], were found to be responsible only for posttransplant infections in our series. *A. baumannii*, a usually multidrug-resistant pathogen [24], has been unfortunately uncommonly found in posttransplant infections [25]–[27]. In agreement with other transplant series [28]–[31], a substantial number of Gram-positive microbes normally considered cutaneous flora were found to be pathogens in ours, and this is not surprising in view that all of the immediate posttransplant recipients were indwelling central-venous catheter and/or other draining catheter/tube of which the exit potentially served as a portal of entry for bacteria normally inhabit in skin.

Bacterial infections in general and bacteremia in particular have been reported to be the major causes of posttransplant mortality [19], [32]–[34]. Our data highlight the importance of a timely alertness to a posttransplant infection and an immediate effective empirical antimicrobial coverage for potential pathogens in ACLF patients. Remarkably, impaired inflammatory response resulting from compromised immunity may clinically attenuate the otherwise marked signs/symptoms of invasive infections in liver transplant recipients [19]–[22], and these patients should therefore be closely monitored and evaluated by experienced clinicians after liver transplantation, preferably by a dedicated infectious-disease specialist.

Limitations of this study include potential biases inherent to the retrospective study and small sample size. Nevertheless, our data suggested that an adequately treated pretransplant infection do not pose a significant risk for clinical outcomes including posttransplant fatality in recipients in adult-to-adult LDLT for ACLF.

## References

[1] Chan AC, Fan ST, Lo CM, Liu CL, Chan SC, et al (2009) Liver transplantation for acute-on-chronic liver failure. Hepatol Int 3: 571–581. 19680733 10.1007/s12072-009-9148-8 PMC2790588

[2] Varghes J, Gomathy N, Rajashekhar P, Venugopal K, Olithselvan A, et al (2012) Perioperative bacterial infections in deceased donor and living donor liver transplant recipients. J Clin Exp Hepatol 2: 35–41. 25755404 10.1016/S0973-6883(12)60081-4 PMC3940144

[3] Sarin SK, Kumar A, Almeida JA, Chawla YK, Fan ST, et al (2009) Acute-on-chronic liver failure: consensus recommendations of the Asian Pacific Association for the Study of the liver (APASL) Hepatol Int. 3: 269–282. 10.1007/s12072-008-9106-x PMC2712314 19669378

[4] Blei AT, Córdoba J (2001) The Practice Parameters Committee of the American College of Gastroenterology. Hepatic encephalopathy. Am J Gastroenterol 96: 168–176. 10.1111/j.1572-0241.2001.03964.x 11467622

[5] Levy MM, Fink MP, Marshall JC, Abraham E, Angus DC, et al (2003) SCCM/ESICM/ACCP/ATS/SIS International Sepsis Definitions Conference. Intensive Care Med 29: 530–538. 12664219 10.1007/s00134-003-1662-x

[6] Garner JS, Jarvis WR, Emori TG, Horan TC, Hughes JM (1988) CDC definitions of nosocomial infections. Am J Infect Control 16: 128–140. 2841893 10.1016/0196-6553(88)90053-3

[7] Beck KD, Gastmeier P (2003) Clinical or epidemiologic diagnosis of nosocomial pneumonia: is there any difference? Am J Infect Control 31: 331–335. 14608298 10.1016/s0196-6553(02)48203-x

[8] Sun HY, Cacciarelli TV, Singh N (2010) Impact of pretransplant infections on clinical outcomes of liver transplant recipients. Liver Transplantation 16: 222–228. 20104499 10.1002/lt.21982

[9] Hindler J (1998) Antimicrobial susceptibility testing. In: Isenberg HD ed. Essential procedures for clinical microbiology. Washington, DC: ASM Press. 205–255 p.

[10] Clinical and Laboratory Standards Institute (2007) Performance standards for antimicrobial susceptibility testing. Approved standard M100–S16. Clinical and Laboratory Standards Institute, Wayne, PA.

[11] Chen CL, Chen YS, de Villa VH, Wang CC, Lin CL, et al (2000) Minimal blood loss living donor hepatectomy. Transplantation 69: 2580–2586. 10910280 10.1097/00007890-200006270-00018

[12] de Villa VH, Chen CL, Chen YS, Wang CC, Wang SH, et al (2000) Outflow tract reconstruction in living donor liver transplantation. Transplantation 70: 1604–1608. 11152222 10.1097/00007890-200012150-00011

[13] de Villa VH, Chen CL, Chen YS, Wang CC, Lin CC, et al (2003) Right lobe living donor liver transplantation-addressing the middle hepatic vein controversy. Ann Surg 8: 275–282. 10.1097/01.SLA.0000081093.73347.28 PMC1422691 12894022

[14] Kamath PS, Kim WR (2007) The model for end-stage liver disease (MELD). Hepatology 45: 797–805. 17326206 10.1002/hep.21563

[15] Garcia-Tsao G (2004) Bacterial infections in cirrhosis. Can J Gastroenterol 18: 405–406. 15190398 10.1155/2004/769615

[16] Berg CL, Gillespie BW, Merion RM, Brown RS Jr, Abecassis MM, et al (2007) Improvement in survival associated with adult-to-adult living donor liver transplantation. Gastroenterology 133: 1806–1813. 18054553 10.1053/j.gastro.2007.09.004 PMC3170913

[17] Chen TC, Lin PC, Chia CY, Ho CM, Chou CH, et al (2010) Infection in liver transplant recipients: analysis of 68 cases at teaching hospital in Taiwan. J Microbiol Immunol Infect 44: 303–309. 10.1016/j.jmii.2011.01.030 21524959

[18] Huprikar S (2007) Update in infectious diseases in liver transplant recipients. Clin Liver Dis 11: 337–354. 17606211 10.1016/j.cld.2007.04.006

[19] Patel G, Huprikar S (2012) Infectious complications after orthotopic liver transplantation. Semin Respir Crit Care Med 33: 111–124. 22447265 10.1055/s-0032-1301739

[20] Xu X, Liu X, Ling Q, Wei Q, Liu Z, et al (2013) Artificial liver support system combined with liver transplantation in the treatment of patients with acute-on-chronic liver failure. PLoS One 8: e58738. 23516546 10.1371/journal.pone.0058738 PMC3597613

[21] Bonnel AR, Bunchorntavakul C, Reddy KR (2011) Immune dysfunction and infections in patients with cirrhosis. Clin Gastroenterol Hepatol 9: 727–738. 21397731 10.1016/j.cgh.2011.02.031

[22] Fishman JA (2007) Infection in solid-organ transplant recipients. N Engl J Med 357: 2601–2614. 18094380 10.1056/NEJMra064928

[23] Magiorakos AP, Srinivasan A, Carey RB, Carmeli Y, Falasas ME, et al (2012) Multidrug-resistant, extensively drug-resistant and pandrug-resistant bacteria: an international expert proposal for interim standard definition for acquired resistance. Clin Microbial Infect 18: 268–281. 10.1111/j.1469-0691.2011.03570.x 21793988

[24] Liu JW, Ko WC, Huang CH, Liao CH, Lu CT, et al (2102) Agreement assessment of tigecycline susceptibilities determined by the disk diffusion and broth microdilution methods among commonly encountered resistant bacterial isolates: Results from the Tigecycline In Vitro Surveillance in Taiwan (TIST) study, 2008 to 2010. Antimicrob Agents Chemother 56: 1414–1417. 10.1128/AAC.05879-11 PMC3294924 22155819

[25] Gouvêa EF, Martins LS, Halpern M, Ferreira AL, Basto ST, et al (2012) The influence of carbapenem resistance on mortality in solid organ transplant recipients with *Acinetobacter baumannii* infection. BMC Infect Dis 12: 351. 23237530 10.1186/1471-2334-12-351 PMC3538523

[26] Mrzljak A, Peric Z, Kovacevic V, Gustin D, Vrhovac R (2010) Rising problem of multidrug-resistant gram-negative bacteria causing bloodstream infections after liver transplantation. Liver Transpl 16: 1217–1219. 20879020 10.1002/lt.22137

[27] Patel G, Perez F, Bonomo RA (2010) Carbapenem-resistant *Enterobacteriaceae* and *Acinetobacter baumannii*: assessing their impact on organ transplantation. Curr Opin Organ Transplant 15: 676–682. 20930636 10.1097/MOT.0b013e3283404373

[28] Kawecki D, Chmura A, Pacholczyk M, Łagiewska B, Adadynski L, et al (2007) Etiological agents of bacteremia in the early period after liver transplantation. Transplant Proc 39: 2816–2821. 18021994 10.1016/j.transproceed.2007.08.048

[29] Linares L, Garcı ´a-Goez JF, Cervera C, Almela M, Sanclemente G, et al (2009) Early bacteremia after solid organ transplantation. Transplant Proc 41: 2262–2264. 19715892 10.1016/j.transproceed.2009.06.079

[30] Sganga G, Spanu T, Bianco G, Fiori B, Nure E, et al (2012) Bacterial bloodstream infections in liver transplantation: etiologic agents and antimicrobial susceptibility profiles. Transplant Proc 44: 1973–1976. 22974885 10.1016/j.transproceed.2012.06.055

[31] Bert F, Larroque B, Paugam-Burtz C, Janny S, Durand F, et al (2010) Microbial epidemiology and outcome of bloodstream infections in liver transplant recipients: an analysis of 259 episodes. Liver Transpl 16: 393–401. 20209598 10.1002/lt.21991

[32] Singh N, Paterson DL, Gayowski T, Wagener MM, Marino IR (2000) Predicting bacteremia and bacteremic mortality in liver transplant recipients. Liver Transpl 6: 54–61. 10648578 10.1002/lt.500060112

[33] Iida T, Kaido T, Yagi S, Yoshizawa A, Hata K, et al (2010) Posttransplant bacteremia in adult living donor liver transplant recipients. Liver Transpl 16: 1379–1385. 21117247 10.1002/lt.22165

[34] Ikegami T, Shirabe K, Matono R, Yoshizumi T, Soejima Y, et al (2012) Etiologies, risk factors, and outcomes of bacterial pneumonia after living donor liver transplantation. Liver Transpl 18: 1060–1068. 22674905 10.1002/lt.23483

